# Awareness of a national initiative to provide free vapes for smoking cessation (Swap to Stop): a survey of adults in England

**DOI:** 10.1093/ntr/ntag077

**Published:** 2026-04-09

**Authors:** Leonie Brose, Vera Buss, Jamie Brown, Ann McNeill, Erikas Simonavičius

**Affiliations:** Department of Addictions, Institute of Psychiatry, Psychology and Neuroscience, King’s College London, London SE5 8BB, United Kingdom; NIHR Policy Research Unit in Addictions, United Kingdom; Department of Behavioural Science and Health, University College London, London WC1E 7HB, United Kingdom; NIHR Policy Research Unit in Addictions, United Kingdom; Department of Behavioural Science and Health, University College London, London WC1E 7HB, United Kingdom; Department of Addictions, Institute of Psychiatry, Psychology and Neuroscience, King’s College London, London SE5 8BB, United Kingdom; NIHR Policy Research Unit in Addictions, United Kingdom; Department of Addictions, Institute of Psychiatry, Psychology and Neuroscience, King’s College London, London SE5 8BB, United Kingdom; NIHR Policy Research Unit in Addictions, United Kingdom; European Union Drugs Agency (EUDA), 1249-289 Lisbon, Portugal

**Keywords:** Vaping, Electronic cigarettes, Smoking Cessation, Surveys, Health inequities, Population health

## Abstract

**Introduction:**

In 2024, England implemented a world-first initiative providing free vapes and behavioral support to encourage smoking cessation, focusing on groups with higher smoking prevalence. This study aimed to assess awareness of the initiative and associations with sociodemographics, smoking, vaping, and past smoking cessation behavior.

**Methods:**

Representative cross-sectional surveys conducted January 2024-December 2025 of adults (aged 16+) who currently smoked or had stopped in the last year in England (*N* = 6950). Associations between awareness and gender, age, ethnicity, socioeconomic position (occupational social grade), region, smoking status, urges to smoke, vaping status, survey time were assessed using weighted percentages with 95% confidence intervals (CI) and multivariable logistic regressions. Multivariable logistic regressions assessed associations with past-year cessation attempts and support used. Post hoc country comparisons using October-December 2025 included Scotland and Wales (*n* = 153).

**Results:**

Overall, 24.1% (95% CI: 23.0-25.2) were aware of the initiative. Awareness was lower among people aged 45-54 [adjusted OR = 0.78 (0.64-0.97)] than among people aged <25 and among Asian, Black, Mixed/Other compared with white ethnicities [adjOR = 0.82 (0.70-0.96)]. Awareness was higher among people reporting slight [adjOR = 1.22 (1.02-1.46)] or moderate [adjOR = 1.27 (1.08-1.50] urges than among those reporting no urges to smoke. Awareness was associated with having used vapes during the last cessation attempt [adjOR = 1.40 (1.09-1.80)]; other characteristics were not significantly associated.

**Conclusions:**

About a quarter of people in England who smoked in the past year were aware of a new smoking cessation initiative, and awareness was linked with the use of vapes for cessation but was not higher among socio-economically disadvantaged groups.

**Implications:**

To reach smoke-free goals, increased smoking cessation is needed; a unique initiative made free vapes and support available to people in England who smoke. While a quarter of the target population had heard about the initiative, coordinated communications may have increased awareness among less advantaged groups with higher smoking prevalence to further improve impact on inequalities.

## Introduction

Smoking remains a leading cause of preventable death, disease^1^^,^^2^ and health inequalities,^3^^,^^4^ causing approximately 64 000 deaths annually in England^5^ and disparities in health outcomes.^4^^,^^6^ Thus, the government aims to make England smoke-free (smoking prevalence ≤5%).^7^^,^^8^

In 2023, cigarette smoking prevalence in England remained at 12% and was substantially higher in more socio-economically deprived groups.^9^ Achieving the smokefree target will require increased smoking cessation. A review^10^ outlined recommendations for reducing smoking prevalence, including vapes for smoking cessation. A Cochrane review has found high-certainty evidence for their effectiveness,^11^ population survey data confirm their widespread use and success,^12^ and local stop-smoking services report high success rates with vapes.^13^ A voucher scheme for vapes combined with behavioral support in a deprived area of England achieved 37% carbon monoxide-validated 4-week smoking cessation.^14^

Building on this evidence, in April 2023 England announced a world-first national “Swap to Stop” initiative^15^ as part of a broader strategy to reduce smoking.^16^ The initiative provided vapes at no cost to local authorities or local partnerships to offer to individuals who smoke, coupled with behavioral support. It aimed to enhance local services and to target locally defined priority populations with higher smoking prevalence, such as routine and manual occupations, people with mental health problems or experiencing homelessness. Training for initiative-related behavioral support was available.^17^ The program aimed to distribute vapes to almost 1 in 5 of all people in England who smoke.^15^ Local authorities were invited to apply to participate in the initiative in October 2023 and could choose allocations of vouchers or vapes, from a wide range of available vapes and liquids (excluding disposable vapes). These were then made available to local services, with vapes likely starting to reach clients from March 2024 and the number of participating areas increasing throughout 2024 and the initiative continuing throughout 2025. The initiative was discussed in the national media when first announced^18–20^ and referenced, for example, around New Year,^21^ but it relied on local promotion with no centralized branding, promotion, or campaigns to raise awareness.

This study aimed to assess the awareness of the Swap to Stop initiative among the target population, specifically:

1. What is the level of awareness of the initiative among adults who smoke or have recently stopped smoking?
2. Is awareness associated with socio-demographic, smoking, or vaping characteristics or time since the initiative started?
3. Is awareness associated with past-year smoking cessation behavior (making a cessation attempt, type of support used)?

## Methods

### Sample selection

Data were from the Smoking and Alcohol Toolkit Study, a cross-sectional survey in Great Britain^22^^,^^23^ using random probability and simple quota sampling to select a new sample of approximately 2450 adults aged ≥16 years each month. Data are collected via computer-assisted telephone interviews and weighted; key variables such as sociodemographic characteristics and smoking prevalence are nationally representative.^22^^,^^24^

We included 24 months (January 2024 to December 2025), which surveyed 60 260 people, of whom 9473 had smoked within the past year. Of those in England (*n* = 7239), 289 provided no information on awareness, leaving an unweighted *n* = 6950 people for analysis. Respondents from Wales and Scotland, where the initiative was not rolled out, were included only in a post hoc analysis of the final 3 months when the awareness question was asked there (*n* = 153 in Scotland and Wales).

### Measures

The pre-registration (https://osf.io/rwm7j) provides the full wording.

The main variable of interest was awareness: “‘Swap to Stop’ is a program to help people stop smoking. It gives participants a free e-cigarette / vape starter kit and behavioural support. Have you ever heard of ‘Swap to Stop’?” Response options were: Yes; No; Don’t know, for analysis recoded into “Aware” (Yes) versus “Not aware” (No and Don’t know).

Socio-demographic variables included age (16-24, 25-34, 35-44, 45-54, 55-64, 65+), gender (men, women, in another way), ethnicity (Asian, Black, Mixed/Other, White; for logistic regressions the first three groups were combined), and region (North, Central, South England, and, where included, Scotland, Wales), and socio-economic group measured as occupational grades (ABC1—more advantaged, C2DE—less advantaged^25^).

Smoking and vaping characteristics included smoking status (currently smoking cigarettes, currently smoking other tobacco, or stopped in the past year; for regression, the first two categories were combined). Urges to smoke (none, slight, moderate, or strong to extremely strong) were assessed as dependence markers and currently vaping (yes, no) was recorded. Smoking cessation behavior included whether respondents had made at least one smoking cessation attempt in the past year and if so, what type of support they had used (vapes, other evidence-based support, or no/other support; for research question 3, collapsed into vapes versus other).

Survey time was split into half years (January-June and July-December for 2024 and 2025).

### Analyses

Analyses were conducted in R Studio and SPSS and include England only unless otherwise mentioned.

1. Weighted percentage with 95% CI aware overall and by gender, age, ethnicity, occupational grade, region, smoking status, vaping status, and survey time.
  A post hoc analysis limited to October to December 2025 calculated this for Scotland, Wales, and England.
2. Multivariable logistic regression with awareness as the outcome and variables as for analysis 1. To avoid small categories, those with missing information on gender or selecting “in another way” (unweighted *n* = 50 and *n* = 115, respectively) or missing information on urges to smoke (unweighted *n* = 218) were excluded and binary versions for ethnicity and smoking status used.
  A post hoc analysis assessed an interaction between occupational grade and survey time.
  A post hoc analysis limited to October to December 2025 used an unadjusted logistic regression to assess Scotland and Wales versus England.
3. Separate multivariable logistic regressions additionally including (1) smoking cessation attempt in the past year (excluding unweighted *n* = 177 with missing information on cessation attempt) and (2) type of support used among those who had made a cessation attempt (unweighted *n* = 2361).

### Ethical approval

Ethical approval was granted by the UCL Ethics Committee (ID 0498/001).

### Changes from pre-registration

The pre-registration listed urges to smoke in the measures, but not in the analysis section and did not specify dichotomization of type of support used for RQ 3. We expanded the timeframe and added post hoc analyses as described.

## Results

Over three quarters were currently smoking cigarettes (77.9%), and another 8.0% other tobacco. Most (73.2%) reported urges to smoke, over a third (35.6%) had made a smoking cessation attempt in the past year, mostly (61.2%) without using vapes. A third (33.1%) were currently vaping.

### Awareness of Swap to Stop and associations

Overall, 24.1% (95% CI: 23.0-25.2) had heard of Swap to Stop, 74.4% (73.2-75.5) had not, and 1.5% (1.3-1.9) were unsure. Awareness was associated with age, ethnicity, and urges to smoke. People aged 45 to 54 were less aware of the initiative than those aged 16 to 24 [adjusted OR = 0.78 (95% CI: 0.64-0.97)]. People from the combined group of Asian, Black, and Mixed/Other ethnicities were less aware than those from white ethnicities [adjOR = 0.82 (95% CI: 0.70-0.96)]. Compared with people reporting no urges to smoke, those reporting slight [adjOR = 1.22 (1.02-1.46)] or moderate [adjOR = 1.27 (1.08-1.50)] urges were more likely to be aware of the initiative. Gender, occupational grade, region, smoking status, vaping status, and survey time were not associated with awareness (Table 1). There was a significant interaction between social grade and survey time, with awareness peaking among ABC1 respondents and reaching its lowest level among C2DE respondents in January-June 2025 [adjOR = 0.59 (0.43-0.81)]. Awareness in the final 3 months of 2025 was not statistically significantly lower in Wales [15.8% (6.2-25.4); OR = 0.68 (0.36-1.30); unweighted *n* = 63] and Scotland [23.9% (14.2-33.6), OR = 0.88 (0.53-1.47); unweighted *n* = 90] than in England [26.5% (23.3-29.7), unweighted *n* = 911].

**Table 1:** Sample description, awareness of Swap to Stop and associations between awareness and characteristics.

**Characteristic**	**Group**	**Proportion in the sample, weighted %**	**Aware of Swap to Stop within this group, weighted % (95% CI)**	**Adjusted odds ratio (95% CI), *P*** ^a^
**Gender**	Men	53.9	23.1 (21.7-24.6)	Ref
	Women	44.0	25.2 (23.5-26.9)	1.01 (0.98-1.23), .11
	In another way	1.5	22.8 (15.1-30.5)	Not included
	Missing	0.6	25.0 (6.0-44.0)	Not included
**Age**	16-24	17.6	24.4 (21.8-27.1)	Ref
	25-34	23.4	26.0 (23.6-28.4)	1.02 (0.85-1.22), .86
	35-44	18.4	24.3 (21.6-26.9)	0.95 (0.78-1.15), .59
	45-54	15.1	20.7 (18.0-23.3)	**0.78 (0.64-0.97), .02**
	55-64	13.5	25.0 (22.1-27.8)	0.92 (0.75-1.13), .40
	65+	12.0	23.0 (20.0-25.9)	0.82 (0.66-1.02), .07
	Missing	<0.1	Not reported	Not included
**Ethnicity**	Asian	5.0	21.9 (17.3-26.4)	**0.82 (0.70-0.96), .01**
	Black	3.5	16.8 (12.2-21.4)	
	Mixed/other	7.3	21.6 (18.0-25.2)	
	White	84.3	24.7 (23.5-26.0)	Ref
**Occupational grade**	ABC1	42.7	23.9 (22.5-25.3)	Ref
	C2DE	57.3	24.3 (22.6-25.9)	1.02 (0.91-1.15), .70
**Region in England**	North	27.1	25.1 (22.9-27.3)	Ref
	Central	29.5	23.2 (21.2-25.2)	0.99 (0.85-1.16), .95
	South	43.4	24.0 (22.4-25.7)	1.00 (0.87-1.15), .97
**Smoking status**	Stopped in the past year	14.1	24.9 (22.0-27.9)	Ref
	Smoking cigarettes	77.9	24.3 (23.0-25.5)	0.90 (0.76-1.08), .26
	Smoking other tobacco	8.0	20.7 (17.2-24.2)	
**Urges to smoke**	None	23.7	22.0 (19.9-24.1)	Ref
	Slight	19.6	25.3 (22.8-27.9)	**1.22 (1.02-1.46), .03**
	Moderate	32.5	25.2 (23.3-27.2)	**1.27 (1.08-1.50), .004**
	Strong to Extremely strong	21.1	23.1 (20.7-25.5)	1.09 (0.90-1.31), .39
	Missing	3.0	27.0 (20.6-33.4)	Not included
**Currently vaping**	No	66.9	24.2 (22.8-25.5)	Ref
	Yes	33.1	24.0 (22.0-25.9)	1.00 (0.88-1.13), .95
**Survey time**	Jan to Jun 2024	24.9	24.2 (22.0-26.4)	Ref
	Jul to Dec 2024	23.6	21.7 (19.5-24.0)	0.87 (0.73-1.03), .10
	Jan to Jun 2025	26.2	23.6 (21.5-25.6)	1.04 (0.89-1.22), .61
	Jul to Dec 2025	25.3	26.7 (24.4-29.0)	1.13 (0.96-1.33), .13
**Smoking cessation attempt in past year** ^b^	No	61.9	23.6 (22.2-25.0)	Ref
	Yes	35.6	25.3 (23.5-27.2)	1.13 (0.99-1.29), .07
	Missing	2.6	19.0 (13.2-24.7)	Not included
**Support used** ^c^	None/other	45.4	23.9 (21.2-26.6)	Ref
	Non-vape evidence-based	15.7	25.0 (20.5-29.6)	
	Vapes	38.8	27.2 (24.0-30.3)	**1.40 (1.08-1.80), .01**

Unweighted *N* = 6950, unless otherwise specified.

^a^Unweighted analysis, *n* = 6569 due to exclusion of missing and third gender group and missing urges to smoke.

^b^Separate regression analysis, unweighted *n* = 6420.

^c^Separate regression analysis, unweighted *n* = 2361 who had made an attempt to stop

Results are bold where CIs for odds ratios do not cross 1.

### Association with past smoking cessation behavior

Awareness was not significantly associated with having made a smoking cessation attempt in the past year, although the odds ratio was above 1 and the *P*-value close to the *P* < .05 level [adjOR = 1.13 (0.99-1.29), *P* = .07, *n* = 6420]. Awareness was associated with using vapes during the last attempt [adjOR = 1.40 (1.09-1.80), *P* = .01, *n* = 2361].

## Discussion

A new initiative to increase smoking cessation was known to about a quarter of those who may benefit from it. Awareness was higher among those with higher dependence, while middle-aged adults and people from minority ethnic groups were less likely to be aware. Awareness did not differ by socioeconomic status; however, trends over time differed. This suggests that the aim of increasing quitting, particularly among priority groups, may need further support; this finding is, however, limited by the data coming from a household survey excluding the most deprived groups. Awareness did not increase with the time since the start of the initiative; this may reflect the lack of any central campaign or branding. While initially, reports in the media may have reached the target population, later awareness depended fully on local activities, which may also sometimes not have used the specific terminology “Swap to Stop” when offering vapes and support to their clients. While awareness appeared descriptively lower in countries of Great Britain without the initiative, this was not statistically supported; however, sample sizes were very small, and people in all constituent countries could have heard about the scheme in national media. There was no conclusive evidence of an association between awareness and having attempted to stop smoking; however, among those who made an attempt, awareness was associated with having used vapes for support, suggesting that at least some of the attempts were supported by vapes obtained via the initiative. Cessation attempts supported by vapes have been shown to be more likely to be successful.^12^

An overall awareness of just under 1 in 4 among people who smoke or recently smoked may seem too low to achieve the aim of having almost 1 in 5 people who smoke participate. However, a population-level analysis^26^ from December 2021 to December 2024 found evidence for an increase in the use of vapes for smoking cessation contemporaneous with the start of the initiative, suggesting that the reach may go beyond those directly or knowingly participating. And Stoptober, a national annually recurring campaign, invested £990 000 for its 2020 iteration in targeted, contextual, and national marketing, with national and local partners amplifying its reach. In a bespoke survey of 1000 people who smoked or had recently stopped, 40% were aware of the brand, and 35% recognized the 2020 campaign.^27^ Given that Swap to Stop had no media campaign or branding, its 24% awareness appears respectable.

The self-reported data may be limited by misunderstandings, recall, or other response bias. Awareness questions are also susceptible to social desirability and acquiescence bias, which may inflate reported levels of awareness by 5 to 15 percentage points^28^^,^^29^ and reduce sensitivity to detect differences. Future surveys could include a non-existent campaign to establish the size of the bias. However, the questions were part of a well-established population survey weighted to ensure representativeness for people aged 16 and over in England. The monthly data collection provides contemporaneous information and a large sample size. However, analysis was not sufficiently powered to detect associations for country due to the much shorter period of data collection; smaller categories for some characteristics also had to be collapsed.

Further research is being conducted to assess the impact of this new initiative on smoking cessation, with a focus on reducing inequalities in smoking.^30^ As it is a unique approach worldwide, we are also researching the experience of implementation and participation. Substantial funds have been invested in the initiative, and while smoking cessation interventions generally are highly cost-effective, information for this specific initiative and different populations will be valuable, and preliminary results are emerging.^31^ Finally, unintended consequences and longer-term health outcomes should be assessed.

## Conclusion

About one in four of people in England who smoked in the past year were aware of Swap to Stop, a new campaign providing vapes and support to increase smoking cessation. Those with higher dependence were more likely to be aware and there was a positive association with having used vapes for smoking cessation. Wider reach among socio-economically less advantaged groups would be desirable.

## Data Availability

Upon acceptance, data will be available on the OSF (https://osf.io/rwm7j) alongside the analysis plan and the question wording.
